# The involvement of peritoneal GATA6^+^ macrophages in the pathogenesis of endometriosis

**DOI:** 10.3389/fimmu.2024.1396000

**Published:** 2024-08-12

**Authors:** Mingxin Shi, James A. MacLean, Kanako Hayashi

**Affiliations:** School of Molecular Biosciences, Center for Reproductive Biology, Washington State University, Pullman, WA, United States

**Keywords:** endometriosis, peritoneal macrophages, GATA6, inflammation, pain

## Abstract

Endometriosis is a chronic inflammatory disease that causes debilitating pelvic pain in women. Macrophages are considered to be key players in promoting disease progression, as abundant macrophages are present in ectopic lesions and elevated in the peritoneum. In the present study, we examined the role of GATA6^+^ peritoneal macrophages on endometriosis-associated hyperalgesia using mice with a specific myeloid deficiency of GATA6. Lesion induction induced the disappearance of TIM4^hi^ MHCII^lo^ residential macrophages and the influx of increased Ly6C^+^ monocytes and TIM4^lo^ MHCII^hi^ macrophages. The recruitment of MHCII^hi^ inflammatory macrophages was extensive in Mac*^Gata6^* KO mice due to the severe disappearance of TIM4^hi^ MHCII^lo^ residential macrophages. Ki67 expression confirmed GATA6-dependent proliferative ability, showing different proliferative phenotypes of TIM4^+^ residential macrophages in *Gata6^f/f^* and Mac*^Gata6^* KO mice. Peritoneal proinflammatory cytokines were elevated after lesion induction. When cytokine levels were compared between *Gata6^f/f^* and Mac*^Gata6^* KO mice, TNFα at day 21 in *Gata6^f/f^* mice was higher than in Mac*^Gata6^* KO mice. Lesion induction increased both abdominal and hind paw sensitivities. *Gata6^f/f^* mice tended to show higher sensitivity in the abdomen after day 21. Elevated expression of TRPV1 and CGRP was observed in the dorsal root ganglia after ELL induction in *Gata6^f/f^* mice until days 21 and 42, respectively. These results support that peritoneal GATA6^+^ macrophages are involved in the recruitment and reprogramming of monocyte-derived macrophages. The extensive recruitment of monocyte-derived macrophages in Mac*^Gata6^* KO mice might protect against inflammatory stimuli during the resolution phase, whereas GATA6 deficiency did not affect lesion initiation and establishment at the acute phase of inflammation. GATA6^+^ residential macrophages act to sustain local inflammation in the peritoneum and sensitivities in the neurons, reflecting endometriosis-associated hyperalgesia.

## 1 Introduction

Endometriosis is a chronic inflammatory disease and affects approximately 10% of reproductive-aged women, representing ~190 million women worldwide (1, 2). It is associated with debilitating chronic pelvic pain, dysmenorrhea, and dyspareunia that dramatically reduces the quality of life of women (3–6). Endometriosis is commonly classified into four stages according to the revised criteria from the American Society of Reproductive Medicine (rASRM) or American Fertility Society (AFS) based on lesion size, location, and the extent of adhesions (6, 7). However, endometriosis-associated pain does not correlate with the staging system (6, 8, 9). Patients with stage I disease can have severe pain, while stage IV patients can be asymptomatic (2, 10), indicating that several other factors contribute to disease symptoms.

Rigorous prior research suggests that aberrant inflammation contributes to the onset and progression of endometriosis (11–21). Macrophages are considered to be key players in promoting disease progression (14, 20, 21), as abundant macrophages are present in ectopic lesions (22) and elevated in the peritoneal cavity (20, 23, 24). Transcriptionally and functionally dysregulated macrophages can establish an inflammatory environment by secreting cytokines and chemokines, which encourage lesion growth and progression (13, 17, 20, 21, 24, 25) and contribute to endometriosis-associated pain (24, 26, 27). Peritoneal macrophages also contribute to the inflammatory condition by releasing cytokines and growth factors that stimulate lesion infiltration, growth, and vascularization (20, 24, 28, 29). Indeed, depletion of peritoneal macrophages by clodronate liposomes reduced lesion progression (24).

Macrophages are highly heterogeneous immune cells that play various roles in homeostasis, innate immunity, and tissue development and repair depending on the signals from their local environment (30). In the peritoneum, two major subsets of macrophages have been distinguished based on their ontogeny and differential expression of F4/80 and MHCII (31). Large peritoneal macrophages (LPM) expressing high F4/80 and low MHCII, F4/80^hi^ MHCII^lo^, are derived from embryonic progenitors in the yolk sac and are maintained in the peritoneum by self-renewal (32), while some replenishment from bone marrow (BM)-derived monocytes continuously occurs with aging (33, 34). Small peritoneal macrophages stemmed from hematopoiesis in the BM, expressing low F4/80 and high MHCII, F4/80^lo^ MHCII^hi^ (31). When inflammatory stimuli are induced in the peritoneum, large numbers of BM-derived monocytes are recruited and differentiate into monocyte-derived macrophages that secrete proinflammatory cytokines as an immune response (35). Monocyte-derived macrophages are transcriptionally and functionally distinct from tissue-resident macrophages even though they serve to replenish resident macrophages (36, 37). Replenishment or reprogramming of monocyte-derived inflammatory macrophages to resident macrophages depends on the degree of inflammatory stimuli (37). Recent evidence (15) indicates that monocyte-derived macrophages may protect the peritoneum against lesion establishment, whereas endometrial macrophages are pro-endometriosis, suggesting further diverse functions of macrophage contribution in endometriosis pathophysiology.

LPM represent most of the macrophage population in the peritoneum, and GATA6 is selectively expressed in LPM (38, 39). See (21) https://kanakohayashilab.org/hayashi/en/mouse/peritoneal.immune.cells/ A zinc finger transcription factor, GATA6, is known to transcriptionally regulate residential LPM proliferation, differentiation, metabolism, and survival (38–40). LPM are essential as immunosurveillance patrollers in maintaining peritoneal homeostasis, as summarized in (41, 42). In response to inflammatory stimuli such as infection or sterile inflammation, LPM undergo the macrophage disappearance reaction (MDR) at the early phase of the inflammatory reaction, in which LPM disappear from the peritoneum due to LPM death and/or adherence to the mesothelium (41, 42). Fate-mapping studies exhibit that MDR, due to the death of large numbers of GATA6^+^ LPM, induces significant replenishment with BM-derived monocytes (33, 34). GATA6^+^ LPM also adhere to the mesothelium to repair sterile injury on the surface of visceral organs, such as the liver or intestine (43, 44). They generally cannot interact with mesothelium under normal homeostasis as they are free-floating cells (45). In contrast, multiple studies have demonstrated that peritoneal macrophages contribute to disease advancement, such as ovarian cancer progression and metastasis (46–49), as deleting peritoneal macrophages reduces tumor progression and peritoneal metastasis (46, 47). Gastric cancer patients bearing more peritoneal macrophages show poor prognosis (50). GATA6^+^ LPM invade liver metastases directly from the peritoneum and contribute to metastatic tumor growth and recurrence (51). Thus, GATA6^+^ LPM can also become adapted and act as tumor-associated macrophages (41, 42).

In endometriosis, CD14^hi^ peritoneal macrophages, which are the predominant resident population in the peritoneum in humans (52), are increased in women with endometriosis (9). However, CD14^hi^ peritoneal macrophages negatively correlate with the severity of pelvic pain, which is independent of disease outcome (9). We have previously reported that GATA6^+^ LPM were involved in the establishment of the aberrant inflammatory environment in the peritoneum, which was associated with increased levels of cytokines and chemokines in the peritoneum and lesion vascularization and innervation (20), indicating dysregulation of GATA6^+^ LPM are involved in disease pathophysiology. In the present study, we thus examined the role of peritoneal GATA6^+^ LPM following endometriotic lesion induction on the recruitment and differentiation of peritoneal macrophages and how they affect disease progression, inflammation, and endometriosis-associated hyperalgesia using *Lyz2^cre^ Gata6^f/f^* mice, selectively deficient for *Gata6* only in the macrophage lineage to reduce GATA6^+^ LPM (38–40).

## 2 Materials and methods

### 2.1 Animals

**Figure 1:**
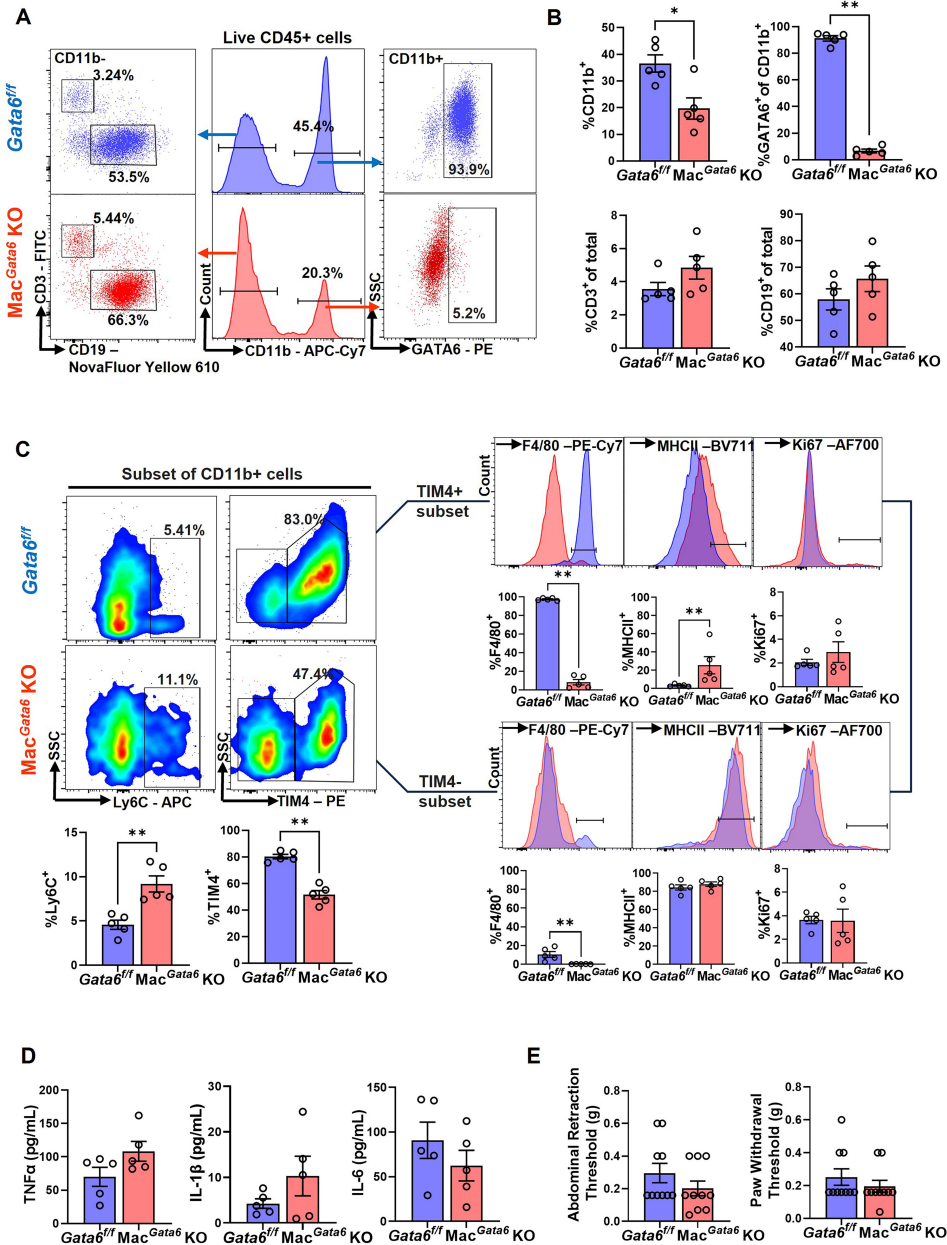
Comparison of peritoneal immune cell profiles between *Gata6^f/f^* and Mac*^Gata6^* KO mice. **(A)** Representative flow plots illustrating the composition of CD11b^+^, GATA6^+^, CD3^+^, and CD19^+^ cells in *Gata6^f/f^* and Mac*^Gata6^* KO mice. **(B)** Proportions of CD11b^+^, GATA6^+^, CD3^+^, and CD19^+^ in *Gata6^f/f^* and Mac*^Gata6^* KO mice. **(C)** Flow cytometric analysis of CD11b^+^ subsets. Bottom left: Proportions of Ly6C^+^ and TIM4^+^ cells from CD11b^+^ subsets. Right panel: TIM4^+^ (top) and TIM4^-^ (bottom) cells were further gated with F4/80, MHCII, and Ki67. Results were shown as representative overlaid histogram plots (*Gata6^f/f^* in blue and Mac*^Gata6^* KO in red) with summarized bar plots below. **(D)** Proinflammatory cytokine levels (TNFα, IL-1β, and IL-6) in the peritoneal fluid were analyzed by IQELISA. **(E)** Abdominal and hind paw hyperalgesia were analyzed using the von Frey test. Data were analyzed by the Mann-Whitney test and shown mean ± SEM (n=5). *P < 0.05, **P < 0.01.

*Gata6*-floxed mice (*Gata6^f/f^*, *Gata6^tm2.1Sad/^*, JAX #008196) and Lysozyme M (Lyz2) Cre mice (*Lyz2^Cr^* ^e^, *Lyz2^tm1(cre)Ifo/J^*, JAX #004781) were purchased from the Jackson Laboratory and *Lyz2^Cre/+^Gata6^f/f^* (denoted as Mac*^Gata6^* KO) and *Lyz2^+/+^Gata6^f/f^* (denoted as *Gata6^f/f^*) mice were generated. All animal experiments were performed at Washington State University according to the NIH guidelines for the care and use of laboratory animals (protocol #6751). Characterizations of peritoneal macrophages in *Gata6^f/f^* and Mac*^Gata6^* KO mice are shown in **Figure 1**.

### 2.2 Mouse model of endometriosis

An experimental mouse model of endometriosis was employed by adopting procedures we have used and described previously with minor modifications (20, 21, 53). Briefly, a ‘menses-like’ event was induced in ovariectomized estradiol-17β- and progesterone-primed donor mice following an established protocol (54). Then, mouse menses-like endometrium scraped from myometrium and cut into fragments (1-2 mm per side) were introduced as the source of syngeneic mouse endometrium (donor: *Gata6^f/f^* mice) via injection (in 0.2 mL PBS) into the peritoneal cavity in untreated naïve mice (recipient: *Gata6^f/f^* or Mac*^Gata6^* KO mice) under anesthesia via inhaled isoflurane. Mac*^Gata6^* KO mice served only as recipient mice, as we have already confirmed that peritoneal macrophages do not arise from donor tissue in this model (20).

### 2.3 Study design

**Figure 2:**
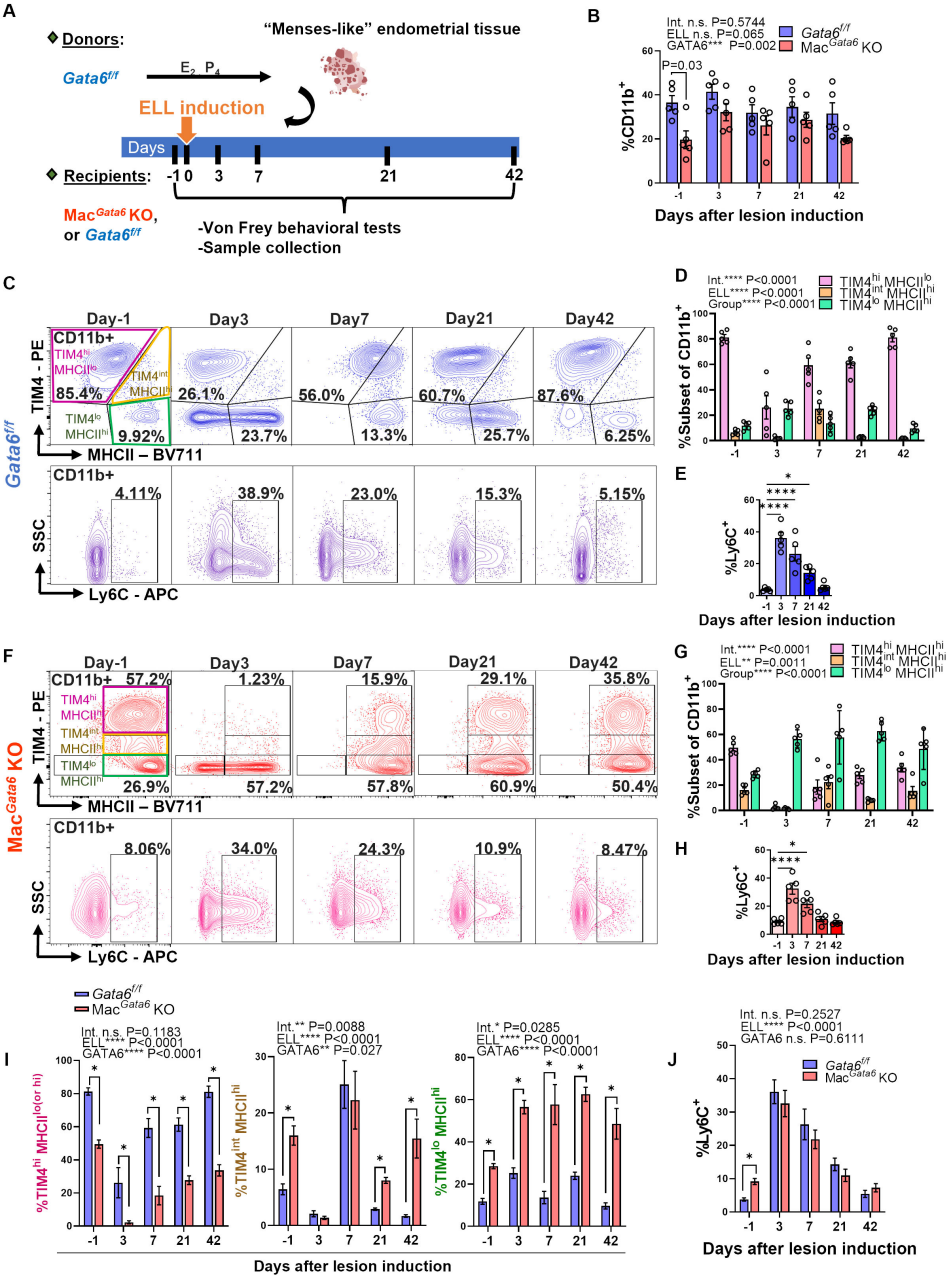
Characterization of peritoneal macrophage profiling following lesion induction in *Gata6^f/f^* and Mac*^Gata6^* KO mice. **(A)** Experimental study design as described in Materials and Methods. **(B)** Graph summarizing flow cytometry data for the proportion of CD11b^+^ cells on Days 3, 7, 21, and 42 after endometriosis-like lesions (ELL) induction (n=5). Two-way ANOVA was performed to determine the main effects of ELL induction (depicted as ELL), GATA6 status (depicted as GATA6), and the interaction effect between the two factors (depicted as Int.). The differences between *Gata6^f/f^* and Mac*^Gata6^* KO mice at each time point were compared by the Multiple Mann-Whitney test. **(C, F)** Representative flow cytometric plots showing the dynamics of macrophages following ELL induction in *Gata6^f/f^* **(C)** and Mac*^Gata6^* KO mice **(F)**. CD11b^+^ cells were further gated by TIM4 and MHCII (top) or Ly6C (bottom) (n=5). Statistical analysis of TIM4^high(hi)^ MHCII^low(lo)^ (pink), TIM4^inter(int)^ MHCII^hi^ (yellow), and TIM4^lo^ MHCII^hi^ (green) cells in *Gata6^f/f^* mice **(D)**, or TIM4^hi^ MHCII^hi^ (pink), TIM4^int^ MHCII^hi^ (yellow), and TIM4^lo^ MHCII^hi^ (green) cells in Mac*^Gata6^* KO mice **(G)** was performed with two-way ANOVA to determine the time-dependent difference after ELL induction (depicted as ELL), the mean difference among cell populations (depicted as Group), and the interaction effect between the two factors (depicted as Int.). Time course analysis for Ly6C^+^ cells in **(E, H)** was conducted by one-way ANOVA with Dunnett’s multiple comparisons test. *P < 0.05, and ****P < 0.0001. **(I)** Time-dependent proportions of TIM4^hi^ (pink), TIM4^int^ (yellow), TIM4^lo^ (green) cells, and **(J)** Ly6C^+^ cells in *Gata6^f/f^* and Mac*^Gata6^* KO mice (n=5). Data were analyzed by two-way ANOVA and Multiple Mann-Whitney tests as described in **(B)**. *P < 0.05. Data are presented as the mean ± SEM.

Endometriosis-like lesions (ELL) were induced in the recipient, *Gata6^f/f^* or Mac*^Gata6^* KO mice, as described above (**Figure 2A**). On Day -1, 3, 7, 21, and 42 (Day 0 = ELL induction), a behavioral test was performed, and mice were then euthanized for sample collections: peritoneal fluid (PF) was recovered by lavage (4 mL x 2 of ice-cold PBS with 3% FBS), ELL, and bilateral lumbar (L4-6) dorsal root ganglia (DRG) were collected for further analysis.

### 2.4 Flow cytometry

Single-cell suspensions of peritoneal exudate cells were used for analyzing immune cell profiles by flow cytometry as described previously (20, 21). Briefly, peritoneal exudate cells were lysed using Red Blood Cell Lysis Buffer (BioLegend) and incubated at room temperature for 20 min with Zombie Aqua™ Fixable Viability dye (Bio-Legend). The cells were blocked on ice for 20 min with Fc Block anti-CD16/CD32 (ThermoFisher) and stained with fluorochrome-conjugated monoclonal antibodies for 1 hour (**Supplementary Table S1**). For GATA6 and Ki67 staining, the cells were fixed and permeabilized with Foxp3/Transcription Factor Staining Buffer Set and Permeabilization Buffer (ThermoFisher). Samples (n=5/group) were acquired with the Attune NxT Acoustic Focusing Cytometer using Attune NxT software (ThermoFisher), and data were analyzed with FlowJo v10.4 software (FLOWJO).

### 2.5 Von Frey test

A standard behavioral (mechanical sensitivity) test was performed before sample collection, as we have performed and others described previously (26, 55, 56). Mice were allowed to acclimate in the testing room for 30 min, and then the von Frey test was performed using von Frey filaments (BIO-VF-M, Bioseb). Filaments were applied 10 times to the skin perpendicular to the lower abdomen and bilateral hind paws. The force in grams (g) of the filament evoking a withdrawal response (50% response count as sensitive) was recorded. Three behaviors were considered positive responses to filament stimulation: 1) sharp retraction of the abdomen, 2) immediate licking and/or scratching of the area of filament stimulation, or 3) jumping. All behavioral tests were performed blindly without describing the identity and details of treatment groups to investigators assessing pain. These data were then analyzed by another independent investigator.

### 2.6 IQELISA

Protein yield from peritoneal fluid was quantitated by BCA assay (Pierce), and TNFα (IQM-TNFA-1), IL-1β (IQM-IL1b-1), and IL-6 (IQM-IL6-1) were further quantified by IQELISA kits (Ray Biotech) according to the manufacturer’s instructions (n=5/group).

### 2.7 Immunohistochemistry

Immunostaining of TRPV1, SP, CGRP, PGP 9.5, and CD68 was performed with cross-sections (5 µm) of paraffin-embedded tissues using specific primary antibodies (**Supplementary Table S1**) and AlexaFluor 488 or 568-conjugated F(ab’) secondary antibody (Molecular Probe) or VECTASTAIN ABC kit (Vector lab). Immunostaining images were acquired by Leica DM4 B microscopy. Cell-specific CD68 positive and total cell numbers were counted by Image J in the area of 0.07244 mm^2^, and the percentage of CD68^+^ cells was shown. PGP9.5 was used as a pan-neuronal marker and was co-stained with TRPV1, SP, or CGRP. TRPV1, SP, or CGRP positive DRG neurons in the section were counted by Image J in the area of 0.07244 mm^2^, and the percentages of TRPV1, SP, or CGRP positive cells per PGP9.5-positive DRG were shown (n=5/group).

### 2.8 Quantitative real-time RT-PCR

Total RNA was isolated from peritoneal exudate cells collected from *Gata6^f/f^* and Mac*^Gata6^* KO mice using TRIzol reagent (Sigma). cDNA templates were synthesized from 1 μg of purified RNA using the High-Capacity cDNA Reverse Transcription Kit (Thermo Fisher). Relative gene expression was examined by SYBR Green Master Mix (Thermo Fisher) using a CFX Opus 96 Real-time PCR system (BioRad) as described previously (20, 21). *Rpl19* was used as the reference gene to normalize mRNA expression levels. Data were analyzed using the 2^-ΔΔCt^ method, and shown as the relative mRNA expression (n=5/group). Specific primer sets were designed using NCBI’s design suite and are shown in **Supplementary Table S2**.

### 2.9 Statistical analysis

Statistical analyses were performed using GraphPad Prism (version 9.5). Mann-Whitney test was used to compare the differences between *Gata6^f/f^* and Mac*^Gata6^* KO mice. One-way ANOVA followed by Dunnett’s multiple comparison tests was used to analyze time-dependent differences within a single group. For grouped data, two-way ANOVA was used to analyze the main effects of ELL induction (depicted as ELL on graphs) and group factor (i.e., GATA6 status, depicted as GATA6; or cell populations, depicted as Group). The interaction effect between the two factors was then calculated (depicted as Int. on graphs). Following two-way ANOVA, to determine the group differences at each time point, multiple Mann-Whitney test with Holm-Šídák correction was used. A P value less than 0.05 was considered to be statistically significant.

## 3 Results

### 3.1 Loss of GATA6 in the peritoneal macrophages

Before studying the impact of ELL induction on macrophage populations, we sought to establish the baseline parameters for immune cell distribution, cytokine levels, and pain sensitivity in mice lacking GATA6^+^ macrophages. In agreement with reported values in three significant papers in 2014 characterizing GATA6^+^ LPM (38–40), we observed an approximately 50% reduction of CD11b^+^ cell populations (macrophages) in the peritoneum in Mac*^Gata6^* KO mice (**Figures 1A, B**). The percentages of CD11b^+^ cell populations in *Gata6^f/f^* mice (36.5 ± 3.2%) and Mac*^Gata6^* KO mice (19.7 ± 4.0 %) were shown in **Figure 1B** (*P* < 0.05). GATA6^+^ CD11b^+^ cells (**Figures 1A, B**; *Gata6^f/f^*, 91.2 ± 2.0 % vs Mac*^Gata6^* KO, 6.7 ± 1.5 %, *P* < 0.01 in **Figures 1B**), or a selective population of F4/80^*hi*^ MHCII^*lo*^ LPM (**Supplementary Figure S1A**; *Gata6^f/f^*, 87.9 ± 0.8 % vs Mac*^Gata6^* KO, 3.8 ± 1.7 %, *P* < 0.05) were greatly reduced in the peritoneum, confirming that the *Lyz2^Cre^* successfully deleted *Gata6*, leading to the loss of GATA6^+^ LPM expressing high F4/80 in Mac*^Gata6^* KO mice (38, 40). In addition, GATA6-regulated genes were also down-regulated in the peritoneal exudate cells of mice lacking GATA6, confirming the loss of GATA6-driven transcriptional factor action (**Supplementary Figure S1B**). However, GATA6 deficiency did not affect CD3^+^ and CD19^+^ cells in the peritoneum (**Figures 1A, B**). When MDR occurs by sterile stimuli, inflammation leads to the recruitment of BM-derived monocytes to replace partially long-lived LPM (32, 34, 57, 58), although remaining LPM proliferate substantially (59). In the persistent loss of GATA6^+^ F4/80^hi^ LPM, an increase in F4/80^lo^ MHCII^hi^ macrophages has been reported (39). We have also observed an increased F4/80^lo^ MHCII^hi^ (**Supplementary Figure S1A**; *Gata6^f/f^*, 7.8 ± 1.0 % vs Mac*^Gata6^* KO, 38.8 ± 2.0 %, *P* < 0.05) and Ly6C^+^ cells in Mac*^Gata6^* KO mice (**Figure 1C**; *Gata6^f/f^*, 4.6 ± 0.5 % vs Mac*^Gata6^* KO, 9.2 ± 0.9 %, *P* < 0.01). Furthermore, F4/80^int^ cells had high levels of MHCII in Mac*^Gata6^* KO mice (F4/80^int^ MHCII^int^, 54.0 ± 3.4 %), which was lowly expressed in GATA6^f/f^ mice (3.7 ± 0.8 %) (**Supplementary Figure S1A**; *P* < 0.05), indicating that GATA6 deficiency induces persistent replenishment from BM in the peritoneum. TIM4^+^ residential macrophages were reduced due to the loss of GATA6^+^ LPM (**Figure 1C**; *Gata6^f/f^*, 80.3 ± 1.7 % vs Mac*^Gata6^* KO, 51.5 ± 3.1 %, *P* < 0.01). As expected, F4/80^+^ cells in Mac*^Gata6^* KO mice were extremely low in either TIM4^+^ macrophage populations (**Figure 1C**; *Gata6^f/f^*, 97.2 ± 0.6 % vs Mac*^Gata6^* KO, 8.2 ± 2.9 %, *P* < 0.01), or TIM4- macrophage populations (**Figure 1C**; *Gata6^f/f^*, 10.6 ± 3.0 % vs Mac*^Gata6^* KO, 0.2 ± 0.1 %, *P* < 0.01). In support of persistent replacement from BM-derived recruited inflammatory macrophages, a high level of MHCII in TIM4^+^ macrophages was observed in Mac*^Gata6^* KO mice (**Figure 1C**; *Gata6^f/f^*, 3.3 ± 0.6 % vs Mac*^Gata6^* KO, 25.5 ± 9.4 %, *P* < 0.01). While a higher basal proliferation rate (SG_2_M phases) of peritoneal macrophages was reported in Mac*^Gata6^* KO mice (39), Ki67^+^ cells in either TIM4^+ or –^ macrophages were not altered by loss of GATA6^+^ LPM in the steady state (**Figure 1C**). Peritoneal cytokine levels, such as TNFα, IL-1β, and IL-6 (**Figure 1D**), as well as abdominal and paw hypersensitivity, were not significantly different between *Gata6^f/f^* and Mac*^Gata6^* KO mice (**Figure 1E**).

### 3.2 Dysregulated peritoneal macrophage resolutions in the absence of GATA6 during endometriosis lesion development

**Figure 3:**
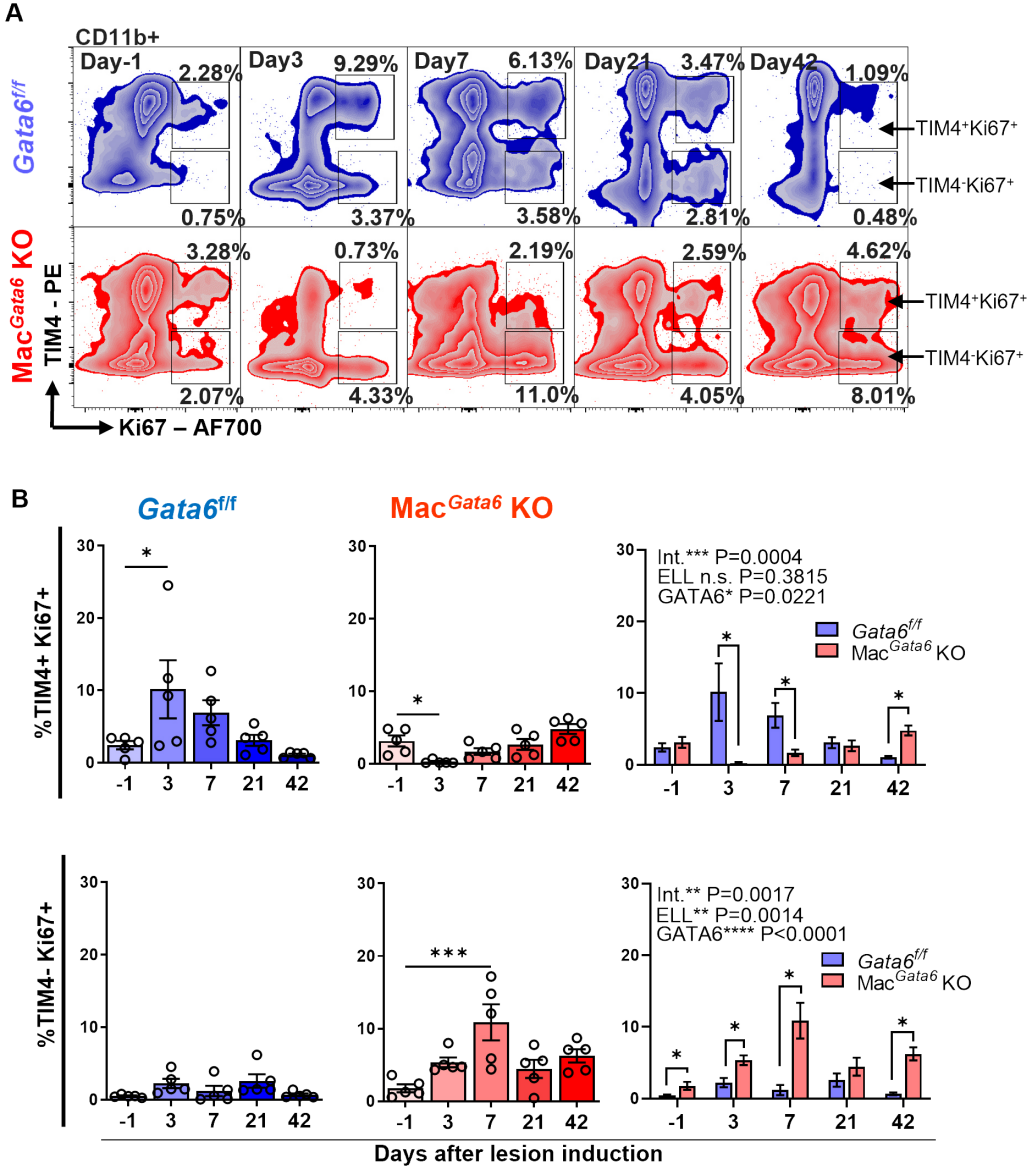
Analysis of TIM4^+^ Ki67^+^ and TIM4^-^ Ki67^+^ cells in CD11b^+^ subsets after ELL induction in *Gata6^f/f^* and Mac*^Gata6^* KO mice. **(A)** Representative flow cytometric plots of CD11b^+^ cells gated with TIM4 and Ki67. **(B)** Bar graphs showing the TIM4^+^ Ki67^+^ (top panel) and TIM4^-^ Ki67^+^ (bottom panel) cell percentages in *Gata6 ^f/f^* in blue, or Mac*^Gata6^* KO in red, or together. Data are presented as the mean ± SEM (n=5). One-way ANOVA, followed by Dunnett’s multiple comparison test, was used to analyze the time-dependent differences in each group. Two-way ANOVA was performed to determine the main effects of ELL induction (depicted as ELL), GATA6 status (depicted as GATA6), and the interaction effect between the two factors (depicted as Int.). The differences between *Gata6^f/f^* and Mac*^Gata6^* KO mice at each time point were compared by the Multiple Mann-Whitney test. *P < 0.05, ***P < 0.001.

We next induced ELL in *Gata6^f/f^* and Mac*^Gata6^* KO mice to examine how the loss of GATA6^+^ LPM affects BM-derived and peritoneal residential macrophages and their resolutions from the stimuli of lesion induction (**Figures 2**, **3**). While the CD11b^+^ population was reduced in Mac*^Gata6^* KO mice prior to ELL induction, within 42 days after ELL induction, there were no significant differences in the CD11b^+^ population between *Gata6^f/f^* and Mac*^Gata6^* KO mice after ELL induction (**Figure 2B**). As shown in **Figures 2C, D**, **Supplementary Figure S2A**, ELL induction in GATA6^f/f^ mice induced the disappearance of TIM4^hi^ MHCII^lo^ residential macrophages 3 days after ELL induction (day -1, 81.3 ± 2.2 % vs. day 3, 26.2 ± 9.1 %, *P* < 0.0001 in **Supplementary Figure S2A**). Concurrently, the influx of increased Ly6C^+^ monocytes was observed 3 days after ELL induction (**Figure 2E**; day -1, 3.8 ± 0.5 % vs. day 3, 36.1 ± 3.6 %, *P* < 0.0001). At the same time, TIM4^lo^ MHCII^hi^ macrophages, as shown in **Figures 2C, D** and **Supplementary Figure S2A**, were significantly elevated (day -1, 11.8 ± 1.4 % vs. day 3, 25.2 ± 2.5 %,*P* < 0.001 in **Supplementary Figure S2A**) following an increase of Ly6C^+^ and TIM4^lo^ MHCII^lo^ cells as the recruitment of inflammatory macrophages (**Figures 2C–E**, **Supplementary Figure S2A**) (15, 21, 60). By day 7, MHCII^hi^ inflammatory macrophages exhibited TIM4^int^ MHCII^hi^ phenotypes consistent with inflammatory macrophages. From days 21-42, TIM4^hi^ MHCII^lo^ residential macrophages recovered and repopulated to the baseline distribution (**Figures 2C, D**, **Supplementary Figure S2A**). In contrast, a more significant disappearance of TIM4^hi^ MHCII^hi^ residential macrophages occurred in Mac*^Gata6^* KO mice within 3 days (**Figures 2F, G, I**, **Supplementary Figure S2B**). The percentages of TIM4^hi^ MHCII^hi^ macrophages were 49.5 ± 2.5 % at day -1 and 2.3 ± 0.9 % at day 3 as shown in **Supplementary Figure S2B** (*P* < 0.0001). Enhanced recruitment of Ly6C^+^ monocytes (**Figures 2H**; day -1, 9.2 ± 0.9 % vs. day 3, 32.6 ± 8.8 %, *P* < 0.0001), or TIM4^lo^ MHCII^hi^ inflammatory macrophages (**Figures 2F, G**; day -1, 28.5 ± 1.2 % vs. day 3, 56.5 ± 3.2 %, *P* < 0.01 shown in **Supplementary Figure S2B**) was also observed by day 3. Interestingly, the recruitment of MHCII^hi^ inflammatory macrophages was extensive in Mac*^Gata6^* KO mice (**Figures 2F, G, I**), probably due to the severe disappearance of TIM4^hi^ MHCII^hi^ residential macrophages. While repopulation of TIM4^hi^ MHCII^hi^ macrophages continuously occurred in Mac*^Gata6^* KO, the replenishment of TIM4^hi^ MHCII^hi^ residential macrophages was slower than in *Gata6^f/f^* mice (**Figures 2F, G, I**, **Supplementary Figure S2B**). As shown in **Supplementary Figure S2AB**, TIM4^hi^ matured residential macrophages recovered to the baseline by day 21 in *Gata6^f/f^* mice (**Supplementary Figure S2A**; day -1, 81.3 ± 2.2 % vs. day 21, 61.3 ± 4.0 %, N.S.), while replenishment of TIM4^hi^ macrophages was still ongoing at day 42 in Mac*^Gata6^* KO mice (**Supplementary Figure S2B**; day -1, 49.5 ± 2.5 % vs. day 21, 27.8 ± 2.6 %, P < 0.001). Indeed, TIM4^int^ MHCII^hi^ macrophages (unmatured) were elevated at only day 7 in *Gata6^f/f^* mice (**Figures 2C, D**, **Supplementary Figure S2A**), whereas TIM4^int^ MHCII^hi^ macrophages in Mac*^Gata6^* KO mice disappeared within 3 days and then repopulated but remained persistent at day 42 during the resolution phase of resident macrophages (**Figures 2F, G, I**, **Supplementary Figure S2B**). Thus, GATA6 deficiency in LPM limits the ability of TIM4^hi^ residential macrophage repopulation following MDR within 6 weeks due to probably loss of proliferative ability with transcriptional signatures (38, 39) and persistently recruits MHCII^hi^ inflammatory macrophages but fails to acquire a mature resident phenotype. Subsequently, Ki67 expression confirmed GATA6-dependent proliferative ability, showing different proliferative phenotypes of TIM4^+^ residential macrophages (**Figure 3**). After the initial disappearance of TIM4^+^ residential macrophages by ELL induction in *Gata6^f/f^* mice, the remaining TIM4^+^ residential macrophages actively proliferated by self-renewal within day 3 (**Figures 3A, B**). As shown in **Figure 3B**, the percentages of TIM4^+^ Ki67^+^ macrophages were significantly increased on day 3 (10.15 ± 4.0 %) compared to day -1 (2.4 ± 0.6 %). This active proliferation probably contributed to the recovery of the TIM4^+^ residential macrophage population by day 21 (**Supplementary Figure S2A**). In Mac*^Gata6^* KO mice, TIM4^+^ residential macrophages almost entirely disappeared and lost their proliferative ability, although they gradually gained proliferative activity to the baseline (**Figures 3A, B**). In contrast, TIM4^-^ macrophages possess a higher proliferative state in Mac*^Gata6^* KO mice, possibly due to the continuous recruitment of inflammatory macrophages.

### 3.3 Peritoneal inflammation in the absence of GATA6 during endometriosis lesion development

**Figure 4:**
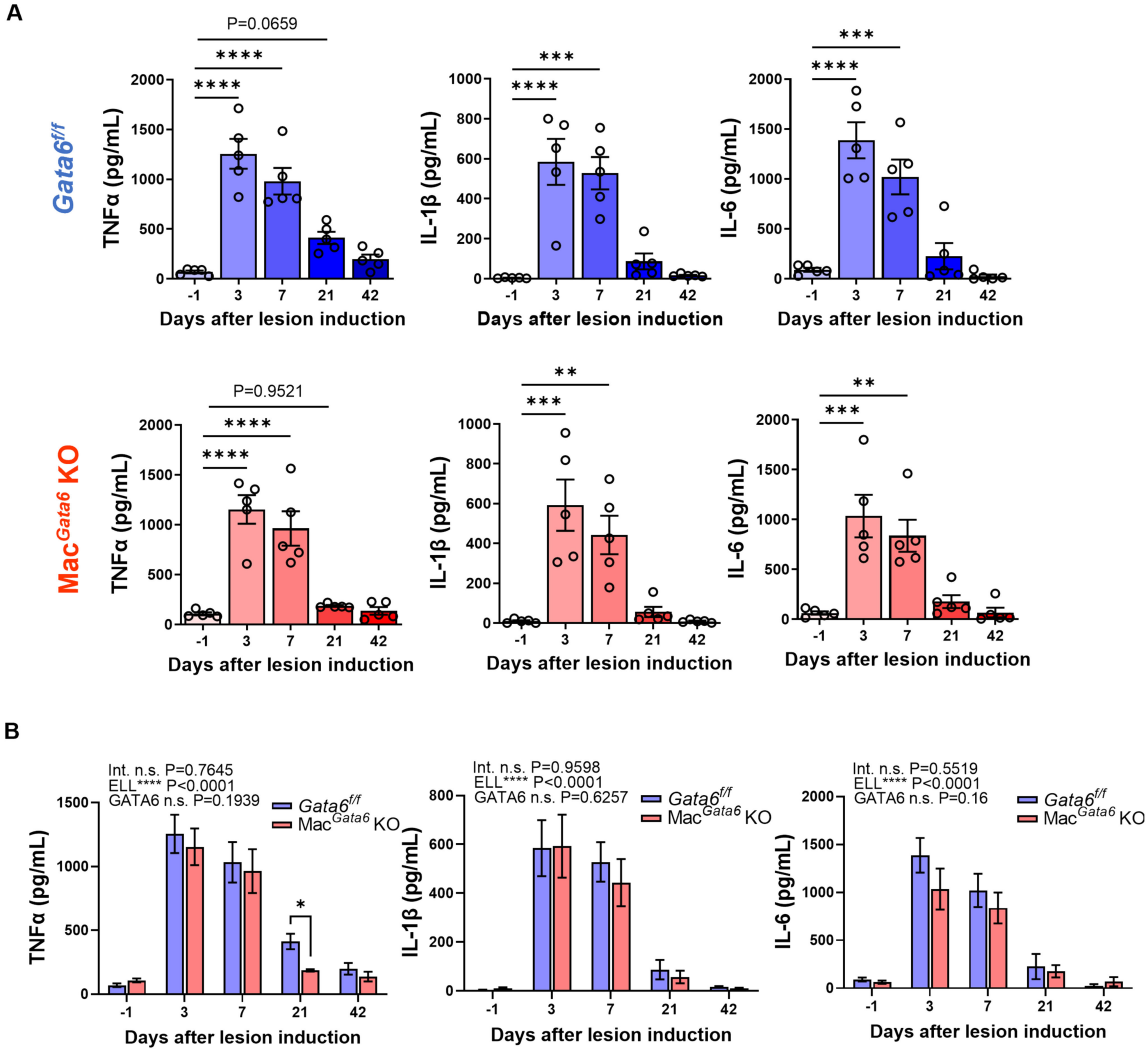
Proinflammatory cytokine levels (TNFα, IL-1β, and IL-6) after ELL induction in *Gata6^f/f^* and Mac*^Gata6^* KO mice. Cytokine levels were shown in a single group **(A)** or grouped plots **(B)**. Data are presented as the mean ± SEM (n=5). **(A)** Data were analyzed with one-way ANOVA followed by Dunnett’s multiple comparisons test. **P < 0.01, ***P < 0.001, and ****P < 0.0001. **(B)** Two-way ANOVA was performed to analyze the main effects of ELL induction (depicted as ELL), GATA6 status (depicted as GATA6), and the interaction effect between the two factors (depicted as Int.). The difference between *Gata6^f/f^* and Mac*^Gata6^* KO mice at each time point was determined by the Multiple Mann-Whitney test. *P < 0.05.

While abundant cytokines and chemokines have been observed in the pelvic cavity of endometriosis patients (13, 16), TNFα, IL-1β, and IL-6 are considered the key factors involved in maintaining the aberrant peritoneal inflammatory environment, promoting lesion growth, and mediating peripheral sensitization (61–63). Elevated TNFα, IL-1β, and IL-6 levels have been reported in the blood, peritoneal fluids, and/or eutopic and ectopic endometrial tissues of women with endometriosis (13, 64–66). Specifically, the levels of TNFα, IL-1β, and IL-6 are increased in peritoneal macrophages isolated from endometriosis patients (67). Thus, we next analyzed the secretion of proinflammatory cytokines, TNFα, IL-1β, and IL-6, in the peritoneal fluids to assess how dysregulated peritoneal macrophage resolutions in the absence of GATA6 affect cytokine secretions (**Figure 4**). ELL induction time-dependently affected cytokine secretions (**Figure 4A**). Peritoneal TNFα, IL-1β, and IL-6 were elevated immediately after ELL induction on day 3 and remained high on day 7 but returned to the baseline by day 21 in both *Gata6^f/f^* and Mac*^Gata6^* KO mice (**Figure 4A**). When cytokine levels were compared between *Gata6^f/f^* and Mac*^Gata6^* KO mice at each time point, TNFα at day 21 in *Gata6^f/f^* mice was higher than in Mac*^Gata6^* KO mice (**Figure 4B**; *Gata6^f/f^*, 412.1 ± 60.7 pg/ml vs. Mac*^Gata6^* KO, 186.1 ± 8.9 pg/ml, *P* < 0.05). Although MHCII^hi^ macrophages were continuously recruited 3-6 weeks after ELL induction in Mac*^Gata6^* KO mice (**Figures 2F, G, I**), the persistent recruitment of inflammatory macrophages did not accelerate the cytokine levels in the peritoneum.

### 3.4 Endometriosis-associated hyperalgesia and pain-related mediators in the absence of GATA6 during endometriosis lesion development

**Figure 5:**
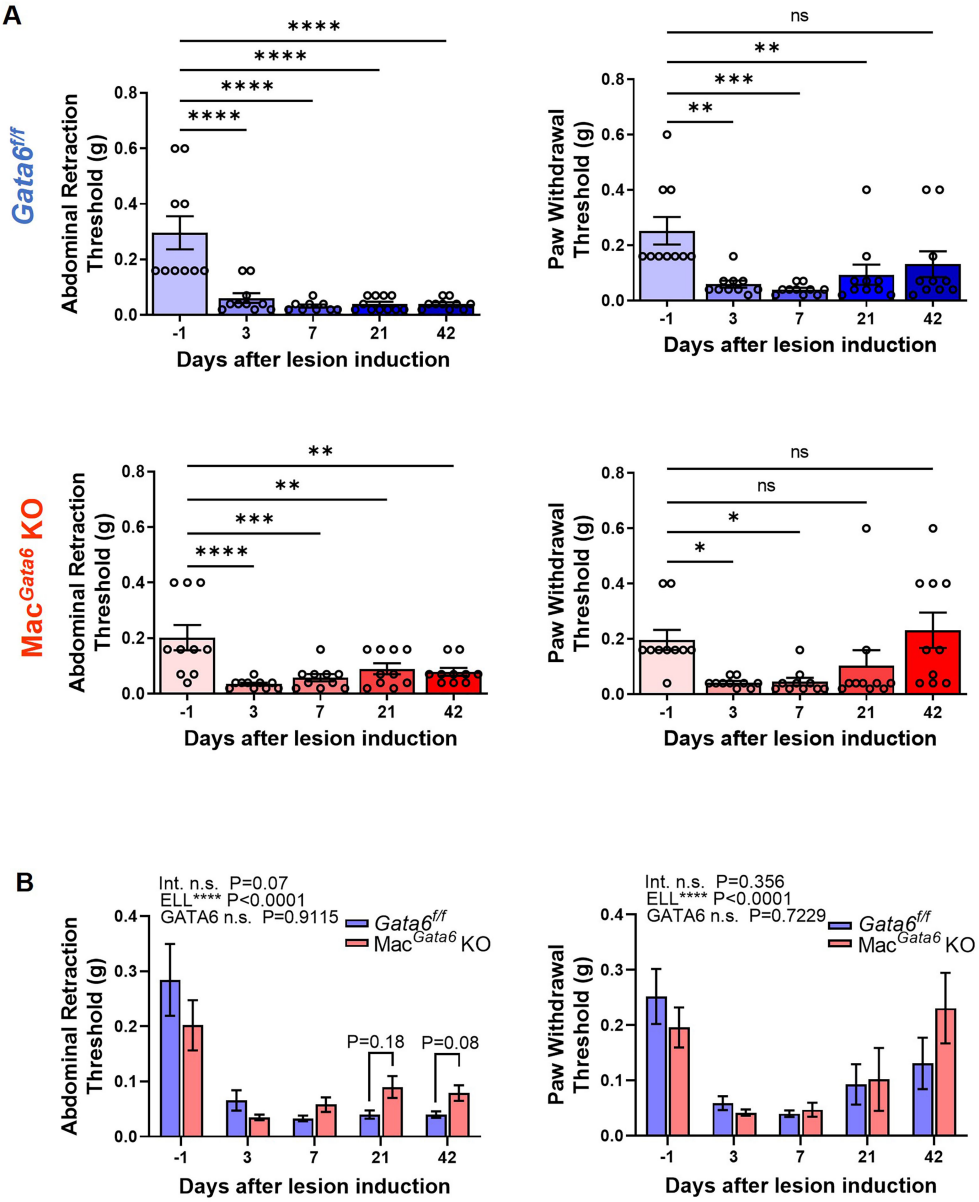
Evaluation of endometriosis-associated hyperalgesia followed by ELL induction in *Gata6^f/f^* and Mac*^Gata6^* KO mice. Abdominal and hind paw withdrawal thresholds were assessed by the von Frey test in *Gata6^f/f^* and Mac*^Gata6^* KO mice. Data are shown by individual group **(A)** and grouped graphs **(B)** as mean ± SEM (n = 10). **(A)** Date were analyzed with one-way ANOVA followed by Dunnett’s multiple comparison test. *P < 0.05, **P < 0.01, ***P < 0.001, ****P < 0.0001. **(B)** Two-way ANOVA was performed to analyze the main effects of ELL induction (depicted as ELL), GATA6 status (depicted as GATA6), and the interaction effect between the two factors (depicted as Int.). The difference between *Gata6^f/f^* and Mac*^Gata6^* KO mice at each time point was determined by the Multiple Mann-Whitney test.

Inflammatory factors in the peritoneum from endometriosis patients can be inflammatory mediators that stimulate neurite outgrowth and endometriosis-associated hyperalgesia (20, 26, 68). We next performed the von Frey test to examine the abdominal and hind paw retraction threshold to determine whether loss of GATA6^+^ LPM affects endometriosis-associated hyperalgesia (**Figure 5**). ELL induction affected time-dependently both abdominal and hind paw sensitivities (**Figure 5A**). *Gata6^f/f^* and Mac*^Gata6^* KO mice withdrew abdominal retraction thresholds with significantly lighter stimuli during the entire study period (**Figure 5A**), even though cytokines levels were only elevated by day 7 (**Figure 4**). *Gata6^f/f^* mice tended to show higher sensitivity after day 21 when the abdominal retraction was compared between *Gata6^f/f^* and Mac*^Gata6^* KO mice at each time point (**Figure 5B**; *Gata6^f/f^*, 0.04 ± 0.01 g vs. Mac*^Gata6^* KO, 0.09 ± 0.02 g at day 21, *P* = 0.18; *Gata6^f/f^*, 0.04 ± 0.01 g vs. Mac*^Gata6^* KO, 0.08 ± 0.01 g at day 42, *P* = 0.08). The hind paw retraction thresholds were sensitive in *Gata6^f/f^* mice until day 21, but after day 7 in Mac*^Gata6^* KO mice, sensitivity returned to the preinduction threshold (**Figure 5A**), whereas no different sensitivity was observed between the groups (**Figure 5B**). The results suggested that *Gata6^f/f^* mice tended to sustain higher endometriosis-associated hyperalgesia for extended periods.

**Figure 6:**
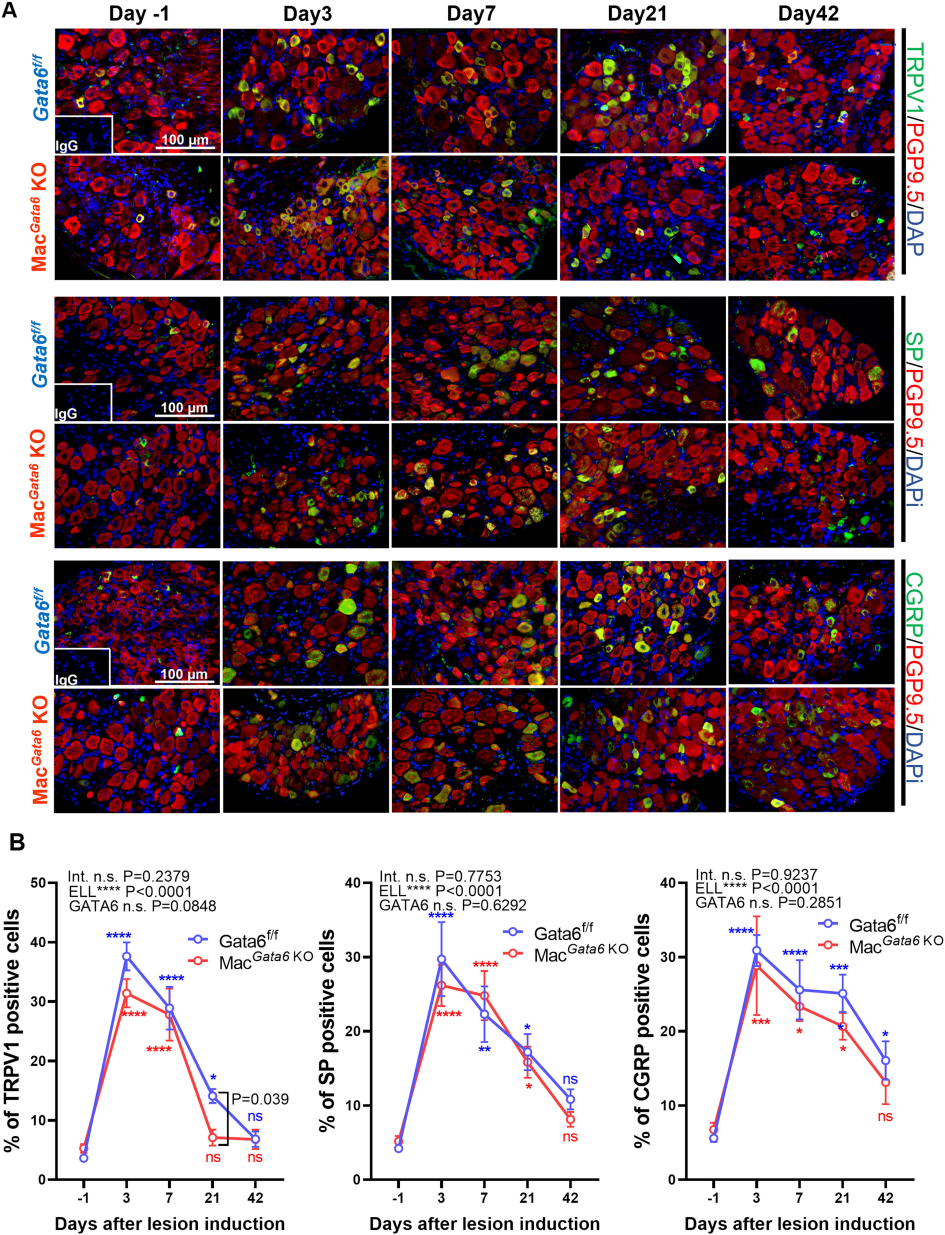
Expression of TRPV1, SP, and CGRP in DRG from *Gata6^f/f^* and Mac*^Gata6^* KO mice after ELL induction. **(A)** Representative images showing DRG sections double stained with TRPV1, SP, or CGRP (green), and PGP9.5 (red), as a marker of DRG cell body. **(B)** Quantification of TRPV1^+^, SP^+^, or CGRP^+^ cells in PGP9.5^+^ cells. Data are shown as the mean ± SEM (n=5). Two-way ANOVA was performed to analyze the main effects of ELL induction (depicted as ELL), GATA6 status (depicted as GATA6), and the interaction effect between the two factors (depicted as Int.). Following two-way ANOVA, Dunnett’s multiple comparison test was used to analyze time-dependent differences compared with day -1 within a group labeled blue (*Gata6^f/f^*) or red (Mac*^Gata6^* KO). The difference between *Gata6^f/f^* and Mac*^Gata6^* KO mice at each time point was determined by the Multiple Mann-Whitney test. *P < 0.05, **P < 0.01, ***P < 0.001, ****P < 0.0001.

Endometriosis-associated hyperalgesia, especially local abdominal sensitivity, should be correlated with the stimuli in the peripheral nerve terminals of nociceptor neurons (69), such as an increase in the expression of transient receptor potential channels, TRPV1. Peripheral nerves can be activated with the increased release of neurotransmitters, such as SP and CGRP. We thus examined TRPV1, SP, and CGRP in the L4-6 DRG, the primary spinal ganglia receiving sensory input from pelvic organs (**Figure 6**). ELL induction increased TRPV1, SP, and CGRP expression time-dependently (**Figures 6A, B**). More TRPV1^+^ DRG were observed until day 21 in *Gata6^f/f^* mice and until day 7 in Mac*^Gata6^* KO mice (**Figures 6A, B**). Higher TRPV1^+^ neurons were detected in *Gata6^f/f^* mice at day 21 compared with those in Mac*^Gata6^* KO mice (**Figure 6B**; *Gata6^f/f^*, 14.1 ± 1.2 % vs. Mac*^Gata6^* KO, 7.1 ± 1.3 % at day 21, *P* = 0.039). SP^+^ DRG were significantly high until day 21 compared with that in the preinduction level in both mouse groups (**Figures 6A, B**). CGRP^+^ DRG were elevated until day 42 in *Gata6^f/f^* mice and until day 21 in Mac*^Gata6^* KO mice (**Figures 6A, B**). Thus, the results support behavior analysis, in which *Gata6^f/f^* mice potentially sustained higher endometriosis-associated hyperalgesia than Mac*^Gata6^* KO mice at later periods.

### 3.5 Endometriosis lesion progression and macrophage presence in the absence of GATA6

**Figure 7:**
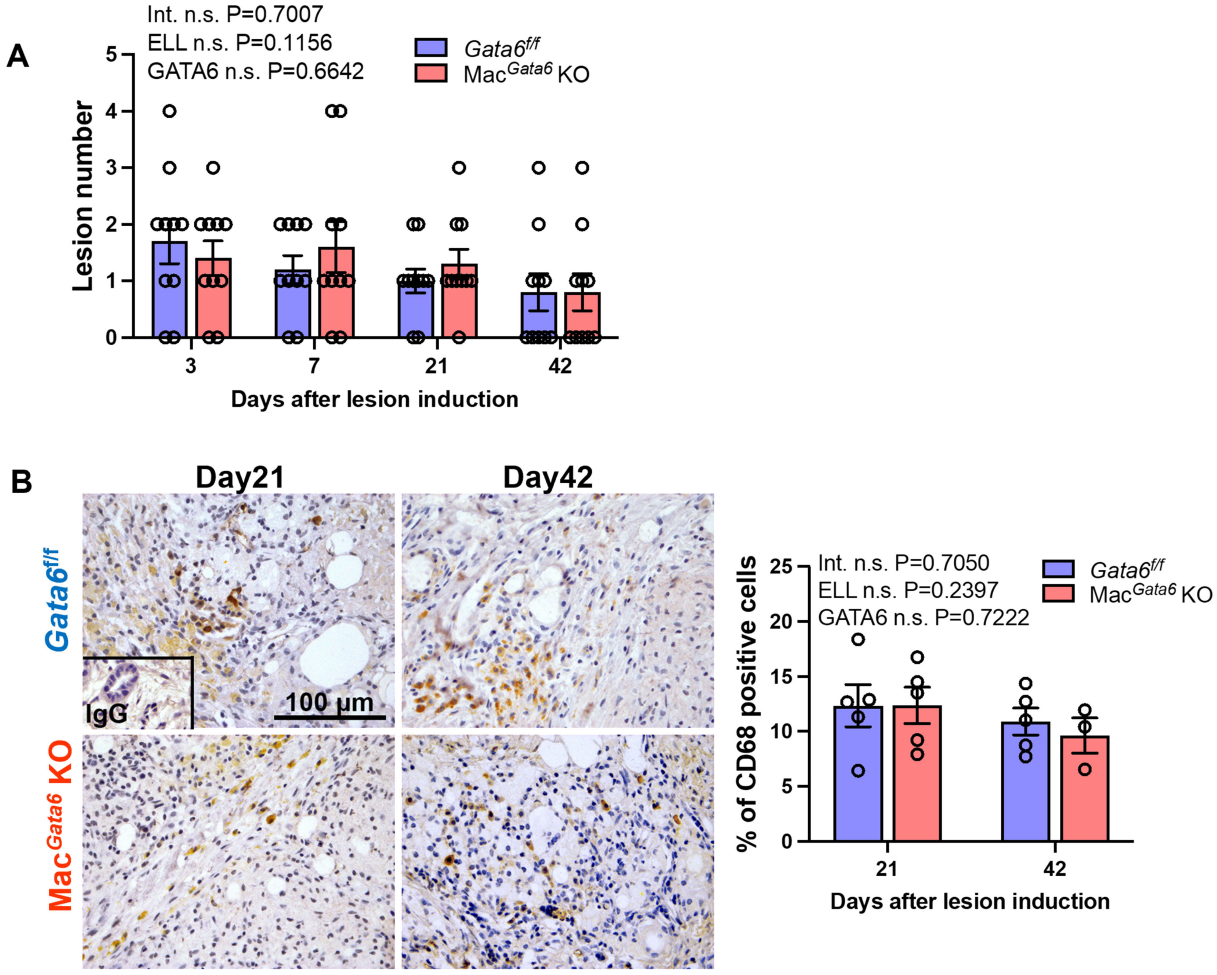
Analysis of ELL in *Gata6^f/f^* and Mac*^Gata6^* KO mice. **(A)** Quantification of lesion numbers in *Gata6^f/f^* and Mac*^Gata6^* KO mice after ELL induction (n=10). **(B)** Representative immunohistochemical images (left) and quantification (right) of CD68^+^ macrophages in the lesions of *Gata6^f/f^* and Mac*^Gata6^* KO mice on days 21 and 42 (n=5). Data are shown as the mean ± SEM. Two-way ANOVA was performed to analyze the main effects of ELL induction (depicted as ELL), GATA6 status (depicted as GATA6), and the interaction effect between the two factors (depicted as Int.). The difference between *Gata6^f/f^* and Mac*^Gata6^* KO mice at each time point was determined by the Multiple Mann-Whitney test. No significant difference was detected.

Lastly, we assessed how GATA6 deficiency affects the progression and growth of endometriosis lesions. Loss of GATA6^+^ LPM did not alter lesion number during the study period (**Figure 7A**). Because we previously reported that peritoneal macrophages can infiltrate ELL (20), we addressed macrophage infiltration in the lesion staining with CD68, a macrophage marker. We only examined macrophage infiltration in the lesion on days 21 and 42, as lesion number, endometriosis-associate hyperalgesia, cytokine levels in the peritoneal cavity, and TRPV1, SP, and CGRP expression in the DRG were not altered by GATA6 deficiency until day 7 in *Gata6^f/f^* and Mac*^Gata6^* KO mice. CD68^+^ macrophages were comparable in both mouse groups at days 21 and 42 (**Figure 7B**). Thus, the absence of GATA6 in the LPM did not affect lesion progression and macrophage infiltration.

## 4 Discussion

Peritoneal macrophages are known to contribute to endometriosis pathophysiology, as dysregulated macrophages increase the secretion of proinflammatory cytokines and reduce phagocytic functions in women with endometriosis (23, 25, 70, 71). Bacci et al. (24) demonstrate that peritoneal macrophages are required for lesion establishment, growth, and vascularization in a mouse model of endometriosis. However, peritoneal macrophages are highly diverse (21, 72), differ in their ontogeny (31), and have transcriptionally and functionally divergent features depending on the signals of the local environment (30). Hogg et al. (15) have reported different characteristics of macrophages depending on their ontogeny in the pathogenesis of endometriosis. They found that monocytopenic mice, which limit monocyte recruitment and subsequently reduce LPM and SPM replenishment, develop increased lesions (15). Furthermore, fewer lesions are developed in mice when embryo-derived LPM are replaced by monocyte-derived LPM, indicating that monocyte-derived LPM protect against endometriosis development (15). In the present study, we focused on the roles of embryo-derived GATA6^+^ LPM, which represent the majority of peritoneal residential macrophages, during ELL establishment and progression. When menses-like tissues are introduced in the peritoneum, it causes acute inflammatory responses. GATA6^+^ LPM are important for the initial uptake where they adhere to the mesothelium to cover organs (41, 42) or die via pyroptosis to release proinflammatory cytokines, such as IL-1β (73), called MDR. If LPM die/disappear, they appear to be replaced by BM-derived macrophages (34). However, when LPM adhere to the walls or mesothelium, they eventually detach and return to the residential LPM in the peritoneum (34). In the present study of *Gata6^f/f^* mice, peritoneal residential LPM (TIM4^+^) disappeared after ELL induction, BM-derived monocytes (Ly6C^+^) were recruited/increased, and MHCII^hi^ macrophages were elevated in the peritoneum. This likely happens immediately after ELL induction, as sterile inflammation induced by intraperitoneal injection with a low dose of zymosan (10 µg) causes MDR and recruited monocytes and inflammatory macrophages in the peritoneum within 4 hours (37). MDR from ELL induction was still observed on day 3 in our study. The significant recruitment of Ly6C^+^ monocytes and MHCII^hi^ macrophages was present until day 21, indicating that LPM pyroptosis with replenishment from BM-derived macrophages also occurs. Supporting the MDR via pyroptosis (34), proinflammatory cytokines, such as TNFα, IL-1β, and IL-6, were significantly elevated in the first week after ELL induction. In contrast, because resident macrophages did not wholly disappear, the remaining macrophages rebounded in number by day 21. In support of this, proliferation in residential LPM was active in the first 3 days and tended to be high even on day 7. Based on the results of increased Ly6C^+^ cells, cytokine levels, and proliferation, “acute inflammatory reactions” continue until 7 days after ELL induction, which is longer than that is induced by a low dose of zymosan (37). During days 21-42, repopulated TIM4^hi^ residential macrophages and BM-derived TIM4^lo^ MHCII^hi^ and TIM4^int^ MHCII^hi^ macrophages returned to the baseline level. LPM in the peritoneum appear to be reestablished by replenishment from BM-derived macrophages and proliferation of remaining residential LPM. Thus, Days 21-42 are likely to be a resolution phase from the inflammatory stimuli (ELL induction). However, we are uncertain whether LPM are fully functional, as monocyte-derived LPM are functionally distinct from embryo-derived residential LPM (37).

Deficiency of GATA6 in the peritoneum induces loss of F4/80^hi^ MHCII^lo^ macrophages (LPM), as previously reported (38, 40). We also observed a significantly reduced macrophage population of highly expressed F4/80. Loss of GATA6 reduced TIM4^+^ resident macrophages as well. Due to the persistent reduction of LPM or resident macrophages, consistent recruitment from BM-derived monocytes and macrophages was seen in our study, as Ly6C^+^ monocytes and MHCII^hi^ macrophages were elevated. In contrast, proinflammatory cytokines were not elevated in the peritoneum of Mac*^Gata6^* KO mice, suggesting that genetically engineered deletion of GATA6 is different from MDR via pyroptosis to release proinflammatory cytokines. When ELL was induced in Mac*^Gata6^* KO mice, severe MDR occurred, as TIM4^+^ resident macrophages almost completely disappeared in addition to the loss of F4/80^hi^ macrophages on day 3. At the same time, MHCII^hi^ macrophages were continuously recruited and maintained at high levels, even on day 42. GATA6 deficiency disrupts peritoneal macrophage proliferation, as GATA6 controls peritoneal macrophage proliferation via its target genes (38, 39). After ELL induction, the remaining residential macrophages are likely less proliferative due to the severe disappearance of TIM4^+^ residential macrophages in Mac*^Gata6^* KO mice on day 3. Instead, recruited TIM4^lo^ MHCII^hi^ macrophages presented higher proliferative activity. However, TIM4^+^ resident macrophages did not fully recover by day 42, indicating that BM-derived TIM4^+^ macrophages probably have a less proliferative ability due to GATA6 deficiency; they are probably distinct from embryo-derived LPM.

Severe MDR also elevated proinflammatory cytokine levels in the peritoneum of Mac*^Gata6^* KO mice, although we only evaluated TNFα, IL-1β, and IL-6, probably via pyroptosis, similar to those in *Gata6^f/f^* mice. Interestingly, TNFα was higher on day 21 in *Gata6^f/f^* mice than in Mac*^Gata6^* KO mice. GATA6^+^ macrophages use an inhibitory pathway to suppress IL-1β processing, a potent activator of the inflammatory cascade in response to inflammatory stimuli, to solve early stages of inflammation (74). It is unclear whether and how GATA6^+^ macrophages are still involved in cytokine secretion after the acute phase of inflammation, and it remains to be examined. However, newly recruited monocyte-derived LPM can protect against lesion development (15). Since GATA6 deficiency enhanced the recruitment of BM-derived macrophages to replenish resident macrophages, they might be involved in the reduction of cytokine levels in the peritoneal cavity. Alternatively, the persistent recruitment of BM-derived macrophages is not directly correlated with cytokine secretion in the peritoneal cavity. On the other hand, GATA6^+^ macrophages can become tumor-associated macrophages to contribute to tumor progression (41, 42), as deletion of peritoneal macrophages reduces tumor progression and peritoneal metastasis in ovarian cancer (46, 47). Peritoneal macrophages expressing higher amounts of a proangiogenic factor, VEGF, are associated with peritoneal metastasis in gastric cancer (50, 75). GATA6^+^ LPM can invade liver malignant tumors and contribute to metastatic tumor growth and recurrence (51). In the mouse model of endometriosis, >25% of macrophages in the lesions are LPM in the peritoneum, indicating that significant trafficking of LPM into the endometriotic lesion might be favorable to maintaining chronic inflammation and disease progression (15).

We showed that the GATA6-deficient peritoneal macrophages did not affect abdominal and paw hyperalgesia in a steady state. When ELL was induced, both *Gata6^f/f^* and Mac*^Gata6^* KO mice immediately exhibited increased endometriosis-associated hypersensitivity in the abdomen on day 3 and sustained until day 42. Increased expression of pain-related mediators supports the result of endometriosis-associated hyperalgesia that was sustained for nearly 6 weeks. However, the sensitivity in the abdomen tended to be severe in *Gata6^f/f^* mice on days 21-42, when most peritoneal macrophage replenishment was resolved. In support of local abdominal sensitivity in *Gata6^f/f^* mice, TRPV1 expression was higher on day 21. Systemic paw sensitivity was significantly high in *Gata6^f/f^* mice until day 21, whereas it was no longer significant on day 21 in Mac*^Gata6^* KO mice. The results suggest that ELL induce acute inflammation in the peritoneum, immediately sensitizing the pain-related neurons. While the resolution of the immune cell population from the stimuli of ELL induction is relatively fast, endometriosis-associated hyperalgesia is sustained longer because the stimuli likely further sensitize pain-related neurons.

Mac*^Gata6^* KO mice showed slightly less hyperalgesia, which can be supported by TNFα level in the peritoneum and TRPV1 expression in the DRG. Hogg et al. (15) demonstrate that monocyte-derived peritoneal macrophages are anti-endometriosis to protect the cavity from lesion development. Mac*^Gata6^* KO mice constantly recruited SPM (monocyte-derived macrophages) due to the deficiency of GATA6 and further enhanced the replenishment of residential macrophages after ELL induction. Thus, large numbers of BM-origin monocyte-derived macrophages exist in the peritoneum. Indeed, TIM4^int^ macrophages differentiated from monocytes are significantly higher in Mac*^Gata6^* KO mice on days 21-42. Therefore, although its mechanism is unclear, monocyte-derived macrophages might also protect against inflammatory stimuli during the resolution phase of inflammation. However, increased TIM4^int^ macrophages were observed in both *Gata6^f/f^* and Mac*^Gata6^* KO mice, suggesting that GATA6^+^ macrophages do not affect earlier lesion development, including establishment. As a supporting result, the lesion numbers were not altered at any stage.

Gibson et al. (9) have reported a human study about endometriosis and peritoneal macrophages. The group shows that CD14^hi^ peritoneal macrophages considered resident peritoneal macrophages in humans (52) are significantly higher than CD14^lo^ peritoneal macrophages in women with endometriosis (9). CD14^hi^ peritoneal macrophages negatively correlate with pain score, whereas CD14^lo^ peritoneal macrophages are positively correlated, independent of endometriosis diagnostic outcome at laparoscopy (9). They assume CD14^hi^ and CD14^lo^ in humans to be equivalent to LPM and SPM in mice (76). However, the authors have noted that CD14^hi^ macrophages likely account for BM-derived macrophages, as monocyte-derived LPM (CD14^hi^ macrophages) are not distinguished. The group concludes that pain symptoms do not correlate with disease extent, as pain scores do not differ among patients at the rASRM stages, indicating inflammatory or immune cell profiles might be a better predictor of endometriosis pain than lesion extent identified during laparoscopy (9).

Evolutionarily, retained body cavities protect internal organs from the external toxic environment, and cavity macrophages contribute to various immune responses (42). LPM maintain peritoneal cavity homeostasis, providing the first line of defense mechanisms (41, 42). Rigorous studies present the vital roles of LPM in repairing injuries and controlling infections by GATA6-driven transcriptional program (41, 42). In the present study, we used a mouse model of endometriosis to study the role of GATA6 resident macrophages following the resolution of inflammatory stimuli (lesion induction). The results suggest the involvement of peritoneal GATA6^+^ macrophages in the peritoneal inflammatory environment, including the reprogramming of monocyte-derived macrophages. Because GATA6 deficiency induced the extensive recruitment of monocyte-derived macrophages and enhanced the replenishment of residential macrophages after ELL induction, they might protect against inflammatory stimuli, especially during the resolution phase. Therefore, GATA6^+^ LPM might be favorable toward sustaining local inflammation in the peritoneum and sensitivities in the neurons, reflecting potentially endometriosis-associated pain during the process of resolution of inflammatory stimuli. Retrograde menstruation causes massive inflammatory responses in the peritoneum. However, menstrual cycles repeatedly occur in women; each retrograde menstruation induces composite inflammation in the pelvic cavity, and unsolved inflammation is expected to worsen to develop chronic conditions further. It might be necessary to induce multiple inflammatory stimuli (multiple ELL induction) to understand the role of residential macrophages and their resolution process in the peritoneum associated with endometriosis-associated hyperalgesia.

## Data Availability

The original contributions presented in the study are included in the article/**Supplementary Material**. Further inquiries can be directed to the corresponding author.

## Supplementary material

Supplementary Figure 1 Verification of *Gata6* depletion in peritoneal macrophages. **(A)** CD11b^+^ cells were gated with F4/80 and MHCII to identify LPM, IM, and SPM. SPM, small peritoneal macrophage; IM, intermediate macrophage; LPM, large peritoneal macrophage. Two-way ANOVA was performed to analyze the mean difference of cell populations (depicted as Population), GATA6 status (depicted as GATA6), and the interaction effect between the two factors (depicted as Int.). The difference between *Gata6^f/f^* and Mac*^Gata6^* KO mice was determined by the Multiple Mann-Whitney test. **P* < 0.05. **(B)** The expression of *Gata6*-regulated genes was examined by Real-time RT-PCR in peritoneal cells (n=5). The Mann-Whitney test was performed to compare the differences between *Gata6^f/f^* and Mac*^Gata6^* KO mice. Data are shown as the mean ± SEM. **P* < 0.05, ***P* < 0.01.

Supplementary Figure 2 Time course analysis of TIM4 and MHCII gated four cell proportions of CD11b^+^ subsets in *Gata6^f/f^* and Mac*^Gata6^* KO mice after ELL induction. Percentages of TIM4^hi^ MHCII^lo(hi)^, TIM4^int^ MHCII^hi^, TIM4^lo^ MHCII^hi^, and TIM4^lo^ MHCII^lo^ cells in *Gata6^f/f^* mice **(A)** and Mac*^Gata6^* KO mice **(B)** following ELL induction. Data were analyzed by one-way ANOVA followed by Dunnett’s multiple comparison test and presented as mean ± SEM (n=5). **P* < 0.05, ***P* < 0.01, ****P* < 0.001, *****P* < 0.0001.
