# Supplementary figures and images for: The involvement of peritoneal GATA6+ macrophages in the pathogenesis of endometriosis
Source: Front Immunol. 2024 Aug 12;15:1396000. doi: 10.3389/fimmu.2024.1396000 (PMC11348394; doi:10.3389/fimmu.2024.1396000)

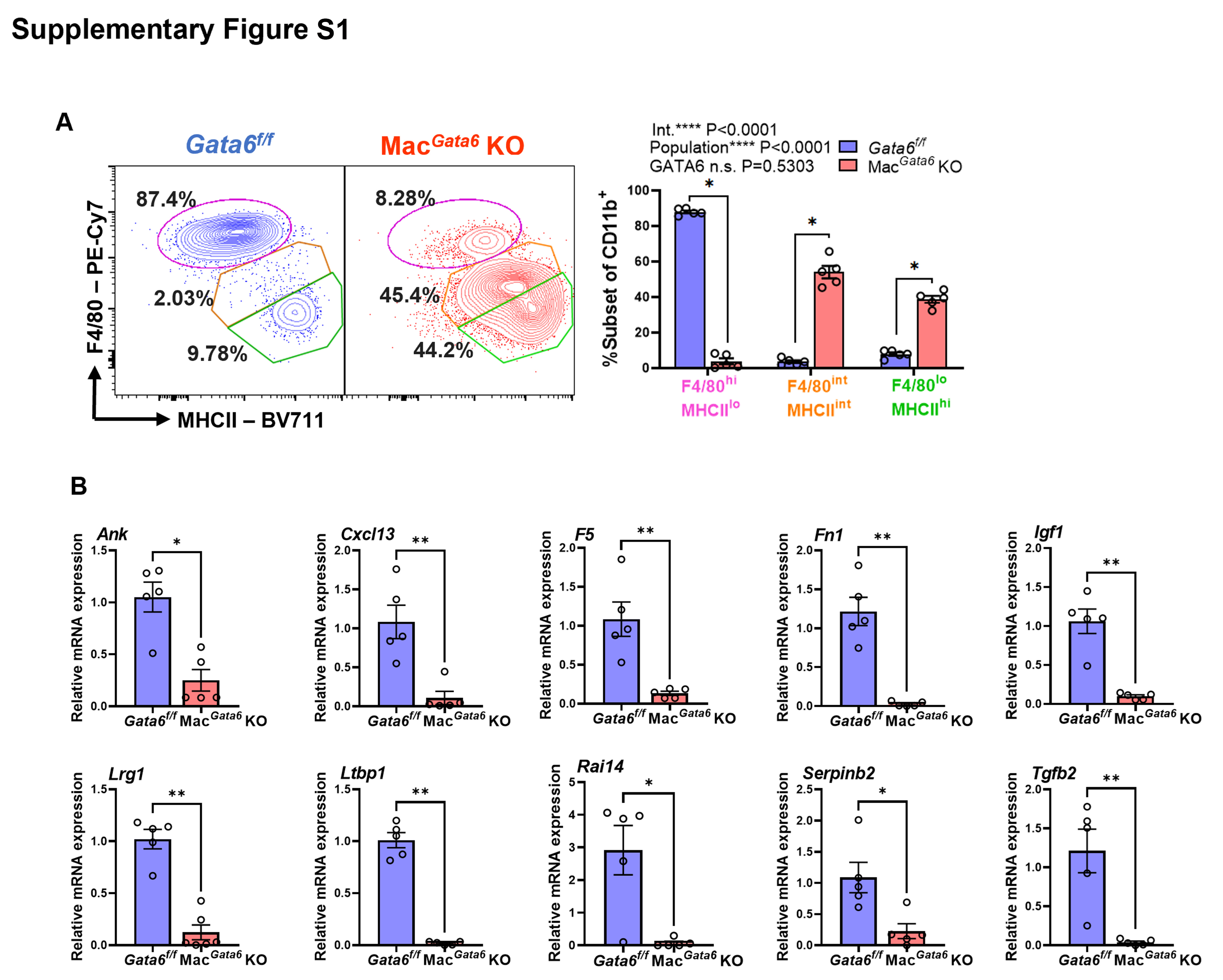

Supplement: Supplementary Figure 1 — Verification of Gata6 depletion in peritoneal macrophages. (A) CD11b+ cells were gated with F4/80 and MHCII to identify LPM, IM, and SPM. SPM, small peritoneal macrophage; IM, intermediate macrophage; LPM, large peritoneal macrophage. Two-way ANOVA was performed to analyze the mean difference of cell populations (depicted as Population), GATA6 status (depicted as GATA6), and the interaction effect between the two factors (depicted as Int.). The difference between Gata6f/f and Mac Gata6 KO mice was determined by the Multiple Mann-Whitney test. *P < 0.05. (B) The expression of Gata6-regulated genes was examined by Real-time RT-PCR in peritoneal cells (n=5). The Mann-Whitney test was performed to compare the differences between Gata6f/f and Mac Gata6 KO mice. Data are shown as the mean ± SEM. *P < 0.05, **P < 0.01. [file Image1.jpeg]

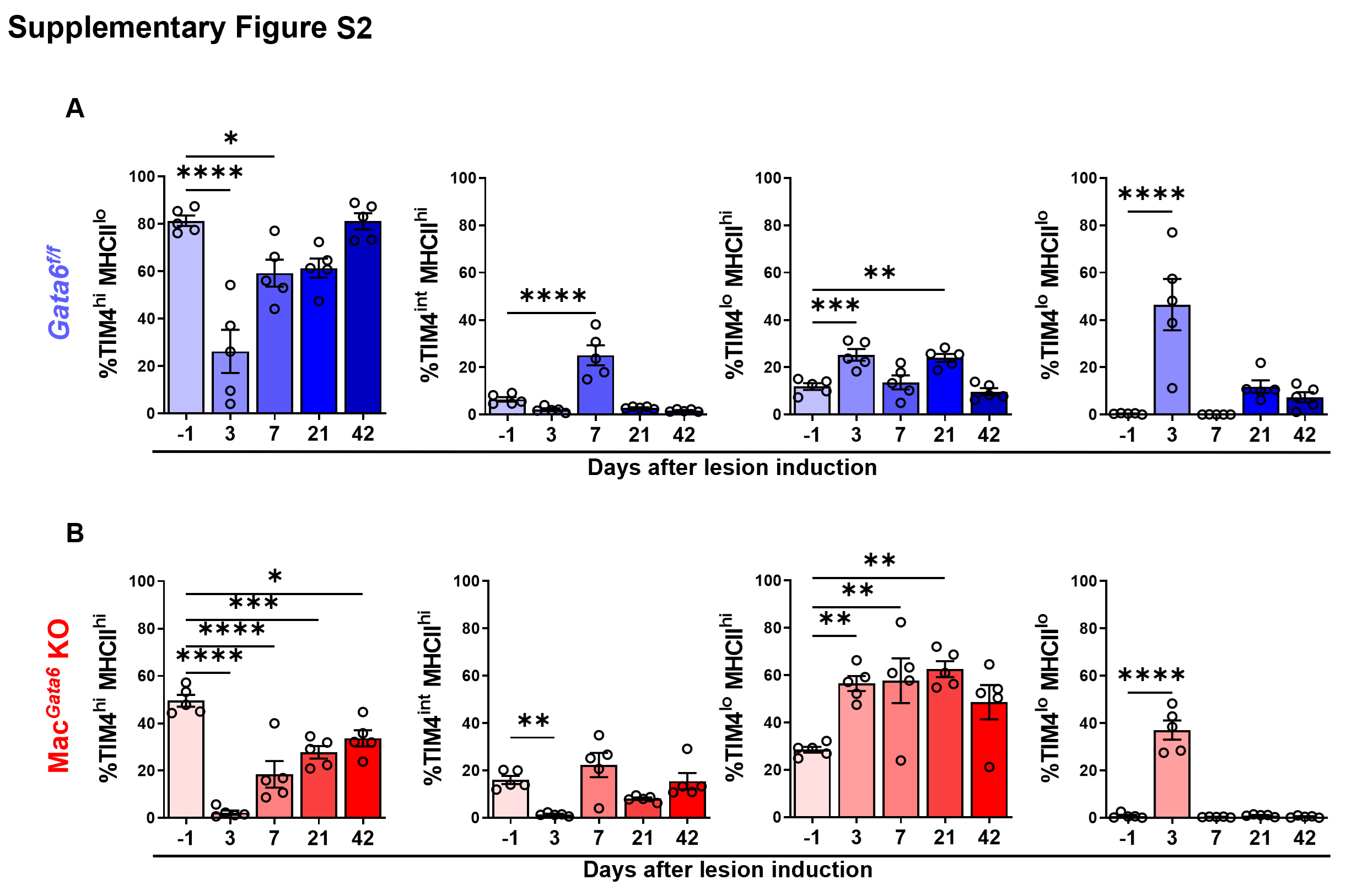

Supplement: Supplementary Figure 2 — Time course analysis of TIM4 and MHCII gated four cell proportions of CD11b+ subsets in Gata6f/f and Mac Gata6 KO mice after ELL induction. Percentages of TIM4hi MHCIIlo(hi), TIM4int MHCIIhi, TIM4lo MHCIIhi, and TIM4lo MHCIIlo cells in Gata6f/f mice (A) and Mac Gata6 KO mice (B) following ELL induction. Data were analyzed by one-way ANOVA followed by Dunnett’s multiple comparison test and presented as mean ± SEM (n=5). *P < 0.05, **P < 0.01, ***P < 0.001, ****P < 0.0001. [file Image2.jpeg]
