# Supplementary material for: The involvement of peritoneal GATA6+ macrophages in the pathogenesis of endometriosis
Source: Front Immunol. 2024 Aug 12;15:1396000. doi: 10.3389/fimmu.2024.1396000 (PMC11348394; doi:10.3389/fimmu.2024.1396000)
Supplement: Supplementary file 3 [file Table1.docx]

| **Antibody** | **Clone** | **Conjugate** | **Company** | **Catalog#** | **Application** |
| --- | --- | --- | --- | --- | --- |
| CD11b | M1/70 | APC-Cy7 | BD Biosciences | 557657 | Flow |
| CD19 | 1D3 | NovaFluor Yellow 610 | eBioscience™ | M004T02Y03 | Flow |
| CD3 | 17A2 | FITC | BioLegend | 100203 | Flow |
| CD45 | 30-f11 | PE-Cy5 | BioLegend | 103109 | Flow |
| CD68 | KP1 | Unconjugated | Abcam | ab955 | IHC |
| CGRP | 4901 | Unconjugated | Abcam | ab81887 | IHC |
| GATA6 | D61E4 | PE | Cell Signaling | 26452S | Flow |
| IA/IE, MHC II | M5/114 | BV711 | BD Biosciences | 563414 | Flow |
| Ki67 | SolA15 | Alexa Fluor 700 | eBioscience™ | 56-5698-82 | Flow |
| Ly6C | AL-21 | APC | BD Biosciences | 560595 | Flow |
| PGP9.5 | PA5-29012 | Unconjugated | Invitrogen | PA5-29012 | IHC |
| Substance P | SP-DE4-21 | Unconjugated | Abcam | ab14184 | IHC |
| TIM4 | RMT4-54 | PE | BioLegend | 130005 | Flow |
| TRPV1 | BS397 | Unconjugated | Abcam | ab203103 | IHC |
| Fc Block CD16/CD32 antibody | |  | Thermo Fisher | 14-0161-82 | Flow |
| Total Antibody Compensation Bead Kit | |  | Thermo Fisher | A10513 | Flow |
| Foxp3 Transcription Factor Fixation/Permeabilization Concentrate and Diluent solutions | |  | Thermo Fisher | 00-5521-00 | Flow |
| Zombie Aqua™ Fixable Viability Kit | |  | BioLegend | 423101 | Flow |

**Supplementary Table S1 Antibodies and Reagents for Flow Cytometry and Immunochemistry**
