# Supplementary material for: The involvement of peritoneal GATA6+ macrophages in the pathogenesis of endometriosis
Source: Front Immunol. 2024 Aug 12;15:1396000. doi: 10.3389/fimmu.2024.1396000 (PMC11348394; doi:10.3389/fimmu.2024.1396000)
Supplement: Supplementary file 4 [file Table2.docx]

**Supplementary Table 2. Primers for qPCR.**

| Accession number | Gene |  | Sequences of forward and reverse primers 5'-3' | Amplicon Length (bp) |
| --- | --- | --- | --- | --- |
| NM_020332 | *Ank* | For | GAAATCCGGGCTGTCTACCC | 141 |
|  |  | Rev | GACAAAACAGAGCGTCAGCG |  |
| NM_018866 | *Cxcl13* | For | CTCTCTCCAGGCCACGGTAT | 201 |
|  |  | Rev | CCATTTGGCACGAGGATTCAC |  |
| NM_007976 | *F5* | For | ACAGCATTTACCCTCACGGG | 173 |
|  |  | Rev | TAGGCACTGGGCATCGTTTT |  |
| NM_010233 | *Fn1* | For | CAAGCCACAGTTTCTGATATTCC | 209 |
|  |  | Rev | TCTGCTCCTGGTTTAATGTTGTT |  |
| NM_010512 | *Igf1* | For | CTGGTGGATGCTCTTCAGTTC | 168 |
|  |  | Rev | CTTCAGTGGGGCACAGTACAT |  |
| NM_029796 | *Lrg1* | For | CTATGGTCTCTTGGCAGCATC | 213 |
|  |  | Rev | AGAATTCCACCGACAGATGG |  |
| NM_001331235 | *Ltbp1* | For | CAAGATGACCTGTGTCGATGTAA | 153 |
|  |  | Rev | GGTGTACAGTAGTTGGGCTTGTC |  |
| NM_030690 | *Rai14* | For | AAGTCGCTTCCCTTACCTTACAC | 163 |
|  |  | Rev | GAGGAAGGGATGTCACTAGCTTT |  |
| NM_001159483 | *Rpl19* | For | TGCCTCTAGTGTCCTCCGC | 237 |
|  |  | Rev | ATCCGAGCATTGGCAGTACC |  |
| NM_011111 | *Serpinb2* | For | GTGCTGAAGAAGCTAGGGAAAA | 194 |
|  |  | Rev | GTTCACACGGAAAGGATAAAGC |  |
| NM_009367 | *Tgfb2* | For | GTCTCAACAATGGAGAAAAATGC | 179 |
|  |  | Rev | CTGGTTTTCACAACCTTGCTATC |  |
|  |  |  |  |  |
